# Multisensory Interactions between Vestibular, Visual and Somatosensory Signals

**DOI:** 10.1371/journal.pone.0124573

**Published:** 2015-04-13

**Authors:** Elisa Raffaella Ferrè, Leif Erik Walther, Patrick Haggard

**Affiliations:** 1 Institute of Cognitive Neuroscience, University College London, London, United Kingdom; 2 Department of Otorhinolaryngology & Head and Neck Surgery, University Medicine Mannheim, University of Heidelberg, Mannheim, Germany; University of Reading, UNITED KINGDOM

## Abstract

Vestibular inputs are constantly processed and integrated with signals from other sensory modalities, such as vision and touch. The multiply-connected nature of vestibular cortical anatomy led us to investigate whether vestibular signals could participate in a multi-way interaction with visual and somatosensory perception. We used signal detection methods to identify whether vestibular stimulation might interact with both visual and somatosensory events in a detection task. Participants were instructed to detect near-threshold somatosensory stimuli that were delivered to the left index finger in one half of experimental trials. A visual signal occurred close to the finger in half of the trials, independent of somatosensory stimuli. A novel Near infrared caloric vestibular stimulus (NirCVS) was used to artificially activate the vestibular organs. Sham stimulations were used to control for non-specific effects of NirCVS. We found that both visual and vestibular events increased somatosensory sensitivity. Critically, we found no evidence for supra-additive multisensory enhancement when both visual and vestibular signals were administered together: in fact, we found a trend towards sub-additive interaction. The results are compatible with a vestibular role in somatosensory gain regulation.

## Introduction

Perception frequently involves interactions between sensory modalities. Sensory signals presented simultaneously in more than one sensory channel tend to be detected more accurately and at lower thresholds than the same signals presented individually [1]. For example, neural responses are enhanced when stimuli in the different sensory modalities are spatially and temporally congruent [2,3,4,5]. Some of these responses may reflect subadditive or superadditive interactions between individual modalities, although the nonlinearity of multisensory responses remains controversial [6,7,8].

Vestibular input contributes to several complex brain functions through its interactions with inputs from other senses. Consistent with this view, vestibular inputs do not project to any primary unimodal cortex. Rather electrophysiological studies have identified a widespread vestibular network whose core area is the parieto-insular vestibular cortex (PIVC) [9,10]. The human homologue of the primate PIVC may not be a single area, so much as a distributed set of regions including the posterior and anterior insula, temporoparietal junction, superior temporal gyrus, inferior parietal lobule, and somatosensory cortices [11,12].

Multisensory neurons coding for visual, vestibular and somatosensory stimuli have been found in the macaque ventral intraparietal area (VIP) [13]. This area is considered homologous to human vestibular areas in the posterior parietal cortex [14]. Evidence suggests that analogous multisensory interactions occur in human perception. For instance, several studies described a convergence between vestibular and visual signals for perception of self-motion [15,16,17].

Multisensory interactions also occur between vestibular and somatosensory signals. Anatomical projections subserving these interactions were inferred from the extensive activation of somatosensory cortices following vestibular stimulation [18]. However, the impact of these interactions for functional perception remains unclear. Vestibular stimulation improves low-level somatosensory perception, such as detection of near-threshold stimuli [19,20,21]. Whereas vestibular-visual interactions can be read as an intermodal combination of cues for a single underlying perceptual dimension of heading direction, the common perceptual content underlying vestibular-somatosensory interactions seems less obvious.

In addition, the behavioural effect of a multisensory interaction depends on the architecture of the projections between the different brain areas involved. For example, Haggard et al. [22] distinguished between *multisensory convergence*, *multisensory modulation*, and *multisensory transformation*, all of which could potentially produce changes in perception. Multisensory convergence involves bimodal neurons which receive inputs from two or more modalities. The visual-vestibular perception of heading discussed above represents an example of multisensory convergence [15,16,17]. The second form of multisensory interaction is modulation by one sensory signal of the gain in a second sensory pathway. The third involves transformation of information from one modality into the spatial reference frame of another. Such transformations involve a change in spatial coding, but may not produce any overall change in neural firing rate. In addition, several studies have reported vestibular-induced changes in activations of classically *unimodal* cortex. Since multimodal neurons are relatively infrequent in these areas (but see [23]) these effects presumably reflect modulation of processing in one modality by another, rather than multisensory convergence of information. For instance, inhibitory visual-vestibular interactions are fundamental in maintaining and controlling gaze [24]. Accordingly, PET studies using artificial vestibular stimulation demonstrated not only an activation of the PIVC but also a decrease in rCBF of the visual cortex [25,26]. Similarly, Bense and co-workers [27] showed bilateral deactivation of the occipital visual cortex induced by vestibular stimulation.

The multiply-connected aspect of vestibular cortical anatomy led us to investigate whether vestibular signals could participate in multi-way interactions with visual and somatosensory inputs. For example, visual and vestibular signals might independently modulate somatosensation. Alternatively, vestibular signals might interact with the effects of vision on somatosensation, by facilitating or suppressing a visual signal that in turn influences somatosensation. A marker of the former arrangement would be that somatosensory effects of combined visual and vestibular stimulation should be predictable from independent effects of vision, and of vestibular stimulation on somatosensation. Conversely, a marker of the latter arrangement would be that results of trimodal stimulation differ from simple superposition of vestibular-somatosensory and visual-somatosensory response patterns.

We investigated bimodal and trimodal interactions between vestibular, visual and somatosensory systems. We used a recently-developed technique that artificially stimulates the vestibular system by a gradual non-contact thermal stimulus of the horizontal semicircular canals via the external auditory canal (Near infrared Caloric Vestibular Stimulation, NirCVS) [28,29,30,31]. A somatosensory detection task was administered immediately after NirCVS. Participants were instructed to detect near-threshold somatosensory stimuli (i.e. digital nerve shocks) that occurred on the left index finger in one half of experimental trials (stimulus present trials). In the other half of the trials the somatosensory stimulus was not present (stimulus absent trials). A simultaneous visual signal occurred close to the finger in half of all trials at random. The visual signal therefore was irrelevant to the participant’s somatosensory detection task. In previous studies, such visual signals were reported to increase somatosensory sensitivity, and also to influence response bias. Specifically, visual signals increased the likelihood of a “yes” response, regardless of whether the somatosensory stimulus was present or not [32,33,34,35]. Based on previous results, we therefore hypothesized an enhancement in somatosensory sensitivity in both bimodal visual-somatosensory and vestibular-somatosensory conditions.

**Fig 1:**
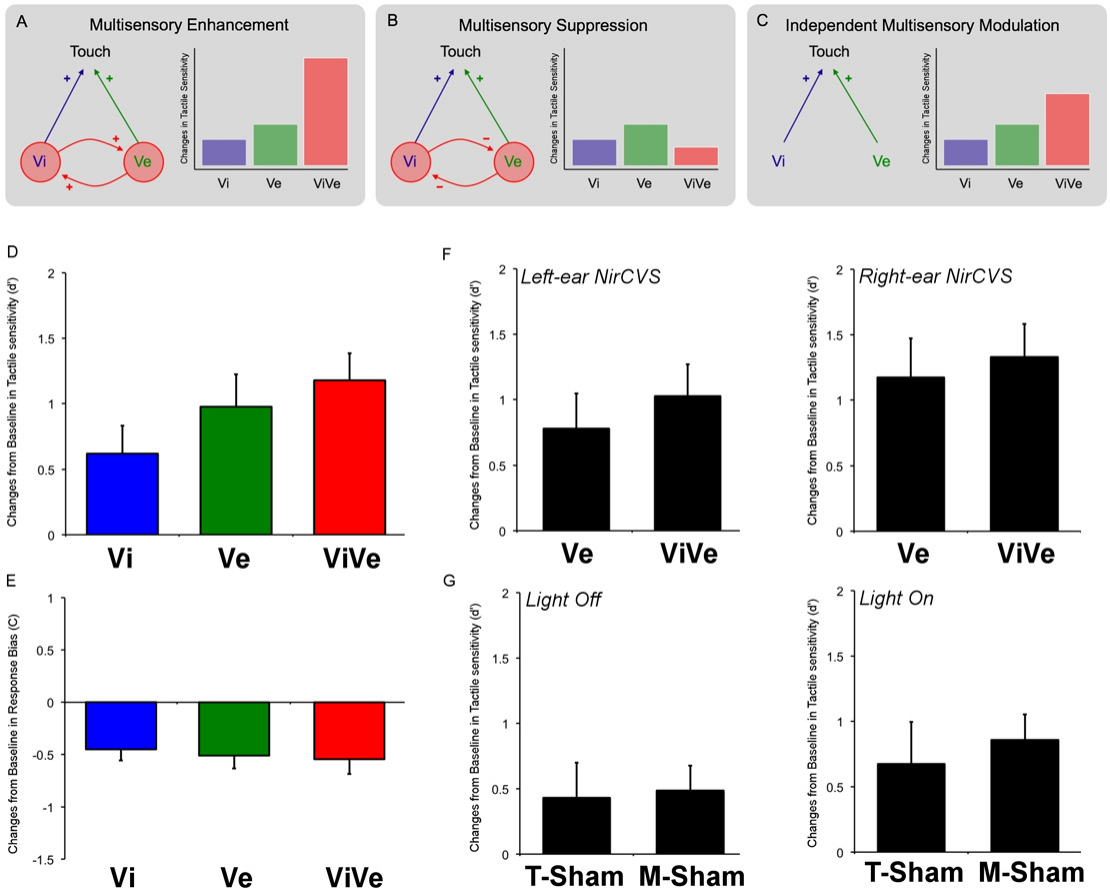
Models and experimental data. (A,B,C) Three schematic models for vestibular, visual and somatosensory interaction, and their possible implications for perceptual sensitivity. Ve: Vestibular condition, Vi: Visual condition, ViVe: combined Visuo-Vestibular condition. Based on previous neuroanatomical, neurophysiological and perceptual results, we focus on three types of multisensory interaction. (A) The three sensory events might produce superadditive multisensory enhancement of somatosensory sensitivity: an illustrative superadditive response is shown by the red bar. (B) Visual and vestibular signals interact through inhibitory connections, as previously described. This model predicts less than additive effects on somatosensory sensitivity: an illustrative example is shown by the red bar. (C) Visual and vestibular inputs might directly, but independently, influence somatosensory sensitivity through separate cross-modal connections leading to simple additive effects (red bar). (D) Changes from Baseline in somatosensory sensitivity in Visual condition, Vestibular condition and in the combined Visuo-Vestibular condition. Mean and standard errors of the mean are reported as function of the experimental condition. Data are presented collapsed across left ear and right ear stimulations. Positive values represent enhanced sensitivity. (E) Changes from Baseline in response bias in Visual condition, Vestibular condition and in the combined Visuo-Vestibular condition. Mean and standard error of the mean are reported as function of the experimental condition. Data are presented collapsed across left ear and right ear stimulations. Negative values in response bias represent a less conservative bias. (F) Changes from Baseline in somatosensory sensitivity in left ear and right ear stimulation in Vestibular condition and in Visuo-Vestibular condition. Mean and standard errors of the mean are reported as function of the experimental condition. (G) Changes from Baseline in somatosensory sensitivity in Thermal Sham (T-Sham) stimulation and Mechanical Sham (M-Sham) stimulation in light off and light on condition. Mean and standard errors of the mean are reported as function of the experimental condition. Data are presented collapsed across left and right ear stimulations.

Simple schematic models of interactions between different sensory systems may be able to generate predictions about somatosensory detection performance. We wanted to compare patterns of interaction that (a) differ strongly in terms of implied connectivity between the different sensory areas, and (b) make plausibly different predictions about likely effects on somatosensory sensitivity. These constraints led us to focus on three specific patterns. First, the three sensory events might combine along the lines of other reported multisensory interactions [1,2,3,4,5], resulting in the superadditive *multisensory enhancement* of somatosensory sensitivity (Fig 1A). This account predicts a trimodal increase in somatosensory sensitivity greater than the sum of the visual-somatosensory enhancement and vestibular-somatosensory enhancement alone, due to an additional facilitatory link between vestibular and visual signals. Second, visual and vestibular signals might interact through inhibitory connections. Inhibitory interactions between visual and vestibular signals have been described for controlling gaze fixation [24,27,36]. A model based on inhibitory connections between visual and vestibular inputs might predict *multisensory suppression* of somatosensory sensitivity (Fig 1B). This model makes the important assumption that inter-areal inhibitory connections reduce functional processing in the target area. However, inhibition in sensory processing does not necessarily impair perception. For example, *local* inhibitory connections within a single sensory representation are important for enhancing perceptual sensitivity, particularly in acuity tasks [37,38]. Moreover, inhibitory integrative neurons in the multisensory parietal cortex were reported to outnumber facilitatory ones [39]. Therefore, multisensory inhibition of perceptual performance must be interpreted with caution. Finally, visual and vestibular inputs might directly, but independently, influence somatosensory sensitivity through separate unimodal-unimodal connections. This model would predict two *independent multisensory modulations* of somatosensory sensitivity, by vestibular and visual input respectively, with the two interactions being simply additive (Fig 1C). Disentangling these three different possibilities provides a novel insight into interactions between these three sensory systems, and may clarify how and where the vestibular system influences somatosensory sensitivity.

## Material and Methods

### 1. Ethics Statement

The experimental protocol was approved by the local ethics committee (University College London) and the study was conducted in line with the Declaration of Helsinki (1995). Participants gave written informed consent to participate in the experiment before inclusion in the experiment.

### 2. Participants

Ten naïve paid participants volunteered in the study (5 male, ages mean ± SD: 25.5 ± 3.14 years). All participants were right-handed as assessed using the Edinburgh handedness inventory [40]. Participants with a history of neurological or psychiatric disorders were excluded prior to taking part in the study. The sample size was set in advance of testing, and was also used as data-collection stopping rule.

### 3. Near infrared Caloric Vestibular Stimulation

To artificially stimulate the vestibular system, we used a recently-developed technique based on a gradual non-contact thermal stimulus which activate the semicircular canals via the external auditory canal [28,29,30,31]. Near infrared Caloric Vestibular Stimulation (NirCVS) is based on the properties of the heat conductance of the bone tissue [28,29,30,31]. Since the maximum depth of penetration of optical radiation into tissues is achieved within the near infrared spectral range from approximately 900 to 1000 nm, near infrared heat radiation is able to penetrate deeply into the bone tissue of the inner ear quickly causing temperature changes in the vestibular organs [28,29,30,31]. NirCVS was delivered with a custom-made device (see for further details [28]). A modified halogen light source (KLQ 150, LOPTEK, Berlin, Germany), with a rated power of a broadband spectrum (λ = 350–2000 nm) in the near infrared range with a peak at about 950 nm. It allowed a total optical output of 2.5 W to be delivered via an ear speculum [28,29,30,31].

NirCVS was performed by positioning the participant’s head 30° backward from the horizontal plane, and 30° away from the stimulated side. This posture aligns the horizontal canals with gravity, and maximises convection currents in the fluid of the semicircular canals. The NirCVS probe was positioned in the auditory canal, and directed at the posterior wall. A trained experimenter held the NirCVS stimulator in place. A mirror inside the probe allowed precise control of the application, location and distance. NirCVS was delivered as a boxcar function with 30 s of duration. In healthy subjects, a VOR (nystagmus reaction) is evoked with broadband near infrared radiation after a stimulation of at least 15 s [28]. Longer stimulations of 30 s lead to stronger effects [28]. A temperature sensor in the device monitored the temperature inside the inner ear and ensured a temperature rise +2°C above the baseline. Immediately after NirCVS was completed, the participant’s head was repositioned to the normal upright position, and somatosensory testing began. Since we were focussing on short-term after effects induced by the stimulation, the actual task was not administered during NirCVS but immediately after.

Functional imaging studies have shown that cortical projections of the vestibular system are asymmetrically organised [41]. These hemispheric differences are related to changes in sensorimotor and cognitive processing following vestibular stimulation in healthy volunteers and in brain-damaged patients. NirCVS was delivered to the right and left ear in separate experimental blocks. To control for non-specific effects induced by NirCVS, we administered two additional control conditions. First, we controlled for the cutaneous thermal sensations evoked during NirCVS, by delivering the near infrared radiation toward the outer ear (Thermal Sham stimulation, T-Sham). Second, we controlled for the unusual feeling of having the stimulator inserted into the auditory canal. We speculated that this would be mediated primarily by cutaneous mechanoreceptors. For this control condition, the stimulator was positioned into the auditory canal, but the near infrared light was not powered on (Mechanical Sham stimulation, M-Sham).

The effectiveness of NirCVS for vestibular stimulation was confirmed by the presence of established vestibulo-ocular responses. EOG recordings showed oculomotor nystagmus with the characteristic contralateral slow phase. Nystagmic fast phases were visually detected and counted for a time window of 90 s after onset of NirCVS (see S1 Fig). The mean nystagmic fast phases, computed as number of saccades per second over the 90 s after onset of vestibular stimulation, was higher for NirCVS (average of left ear and right ear averaged across participants, mean: 0.155 saccades/s, sd: 0.05 saccades/s) compared to after T-Sham (average of left ear and right ear averaged across participants, mean: 0.07 saccades/s, sd: 0.03 saccades/s) (t(9) = 5.403; p<0.001).

A short questionnaire was also administered to describe qualitatively the sensations evoked by NirCVS and sham stimulations. No participant reported experiencing any particular discomfort during NirCVS. Most participants reported a sensation of skin warming during NirCVS. A few participants’ questionnaire responses suggested vestibular sensations, such as “vertigo” (defined as the sensation of whirling motion, either of oneself or of external environment) and “dizziness” (Table 1). These results are not unexpected, and are consistent with a previous report using the same NirCVS method [28].

**Table 1:** Vestibular sensations questionnaire.

	NirCVS		T-Sham		M-Sham	
	Left ear	Right ear	Left ear	Right ear	Left ear	Right ear
Vestibular induced sensations						
Vertigo	3	2	3	0	1	0
Light-headedness	4	4	3	2	3	3
Dizziness	1	1	2	1	1	1
Tendency to fall to the side	0	1	0	1	1	0
Head spinning or turning around	3	2	1	1	2	0
Thermal sensations						
Local somatosensory feeling in the ear	8	8	8	8	6	7
Warm feeling	8	8	9	8	4	2
Side Effects						
Headache	0	0	4	0	1	1
Feeling of Nausea	1	1	2	1	0	0
Blurred vision	2	1	3	1	2	0
Confusion	2	2	3	1	2	1
Slight uncomfortable feeling	5	3	2	3	2	3

Number of participants experiencing vestibular sensations, thermal sensations and side effects during NirCVS and sham stimulations. NirCVS = Near infrared Caloric Vestibular Stimulation; T-Sham = Thermal Sham stimulation; M-Sham = Mechanical Sham stimulation.

### 4. Stimuli and Procedure

Verbal and written instructions about the task were given to participants at the beginning of the session. To reduce the postural consequences of vestibular input and to avoid any motor bias, the experiment was conducted in a comfortable sitting position. Participants were seated in a darkened room in front of a vertical panel that supported and fixed the left hand in the participant’s direct view. A 4 mm diameter red light-emitting diode (LED) was located adjacent to the tip of the left index finger, without covering it. Participants were instructed to fixate the LED close to the left index finger, and to detect faint somatosensory pulses. Somatosensory electrical stimulation was delivered via a pair of ring electrodes placed over the distal phalanxes of the left index finger, with the cathode 1 cm proximal to the anode. Somatosensory stimulation was provided by digital nerve shocks, using a custom-built electrical stimulator, whose current-level and pulse duration were controlled by a computer. Pulse amplitude was held at 10 mA and pulse duration was varied to adjust the charge transferred to the skin, and thus the perceived shock intensity. To identify somatosensory detection thresholds, a staircase procedure was used to estimate the lowest shock intensity at which a somatosensory stimulus could be reliably detected. Pulses of increasing width were applied until participants reported a sensation. Pulse width was successively decreased and then increased again until exactly 5 of 10 stimuli were detected. Next, the pulse intensity obtained with the staircase procedure was tested in a detection block to check that 50% of pulses were reliably detected. This level was considered as working estimate for near-threshold electrical stimulation in each participant.

Our design combined somatosensory, visual and vestibular signals, so that these were all statistically independent. The somatosensory detection task lasted for about 2 minutes and was designed following a signal detection approach [42]. It consisted of a 2 (somatosensory stimulus present/absent) x 2 (visual stimulus present/absent) design, with the following trial types: 15 touch only trials (somatosensory stimulus present and visual stimulus absent); 15 touch and visual trials (somatosensory stimulus present and visual stimulus present); 15 visual only trials (somatosensory stimulus absent and visual stimulus present); and 15 no stimulus trials (somatosensory stimulus absent and visual stimulus absent). Thus, a total of 60 trials were performed in each block. The beginning of each trial was signalled by an auditory warning signal. Somatosensory and visual stimuli (if present) were delivered after an interval of 500 ms from the auditory warning signal. Somatosensory and visual stimuli were temporally coordinated. The LED stayed on for 20 ms, and was easily detectable by the participant. A different tone indicated the end of the trial after a further 500 ms. Participants were required to indicate whether or not they felt the somatosensory stimulus. The somatosensory stimulus was presented at the threshold level previously established. Trial order was randomized, so that participants could not predict somatosensory stimulus and visual stimulus presence. Data for each trial were recorded and analysed later.

One block of the above signal detection task was performed for each of seven experimental condition. These were no NirCVS (Baseline), left ear NirCVS, right ear NIRCVS, and the four sham conditions generated by factorial combination of thermal and mechanical sham, and ear stimulated. Condition order was randomized across participants. Participants were not informed regarding the experimental conditions. An interval of 5 min was present between experimental conditions.

### 5. Data Analysis

Somatosensory detection results were analysed using signal detection analysis. The number of hits (number of somatosensory stimulus present trials in which participants said “yes”), false alarms (number of somatosensory stimulus absent trials in which participants said “yes”), misses (number of somatosensory stimulus present trials in which participants said “no”) and correct rejections (number of somatosensory stimulus absent trials in which participants said “no”) was computed for each experimental condition. We then calculated hit rates [P(“yes” | stimulus present), proportion of hit trials to which subject responded “yes”] and false alarm rates [P(“yes” | stimulus not present), proportion of trials in which the stimulus was absent but the subject responded “yes”]. These were used to obtain estimates of perceptual sensitivity (d’) and response bias (C) using the standard signal detection formulae [42]. Since signal detection does not allow sensitivity and response bias to be computed when the hit rate is 1 or false alarm rate is 0, we applied a standard correction to such values before computing d’ and C estimates. Several corrections have been proposed, but the following, established method was used here [42]. N was considered the maximum number of false alarms. Not counting zero, the smallest false alarm rate is 1/N. Thus, if the measured false alarm rate is 0, the true false alarm rate falls somewhere between 0 and 1/N, so the standard method is to use 1/(2N) instead of zero (i.e. which is the same as saying that we observed half a false alarm). The same reasoning was applied to a hit rate of 1. Instead of using 1, we used 1–1/(2N), where N is now the number of targets.

Four experimental conditions were important for our multisensory inferences: (i) *Baseline* condition (d’ and C estimates based on somatosensory stimulus present trials and somatosensory stimulus absent trials during no vestibular stimulation condition), (ii) *Visual* condition (d’ and C estimates based on somatosensory stimulus present and visual stimulus present trials and light only trials during no vestibular stimulation condition), (iii) *Vestibular* condition (d’ and C estimates based on somatosensory stimulus present trials and somatosensory stimulus absent trials during NirCVS) and (iv) *Visuo-Vestibular* condition (d’ and C estimates based on somatosensory stimulus present and visual stimulus present trials and light only trials during NirCVS).

To estimate effects of concurrent multisensory input on somatosensory detection, we subtracted the sensitivity and response bias in the Baseline condition from those in each stimulation condition, and analysed these difference measures statistically. This subtraction was performed at the single participant level. Subtracting the Baseline condition allowed us to remove individual differences in sensitivity and response bias, highlighting the change in somatosensory detection due to visual and vestibular signals. In a further, separate analysis, we compared both the Vestibular only and the Visuo-Vestibular conditions to our control conditions (i.e. T-Sham and M-Sham), to exclude non-specific, non-vestibular effects of NirCVS on somatosensory detection.

Our hypotheses about visual and vestibular effects on somatosensory detection were analysed by planned contrasts, as follows:

1. Visual signals should influence somatosensory sensitivity [32];
2. Visual signals should induce a less conservative bias [32];
3. Vestibular signals should influence somatosensory sensitivity [11];
4. Concurrent visual and vestibular signals should have interactive effects, such as superadditive multisensory enhancement (Fig 1A), or multisensory suppression (Fig 1B).

Note that, depending on the pattern of interaction found, planned comparison (iv) might only be interpretable with the additional assumption of a monotonic stimulus-response function [43,44].

## Results

Raw mean data for each experimental condition are reported in Table 2.

The sensitivity range in the Baseline condition was between 0.99 and 1.92, consistent with the setting of somatosensory stimulus intensity at a level that avoided floor or ceiling detection performance. Response bias was conservative overall, showing a general reluctance to respond “yes”, irrespective of whether the somatosensory stimulus is present or not (Table 2).

**Table 2:** Sensitivity and Response bias values.

	Sensitivity (d’)		Response Bias (C)	
	Mean	SD	Mean	SD
Baseline (Light off)	1.41	0.37	1	0.36
Visual—Vi (Light on)	2.03	0.72	0.55	0.40
Vestibular—Ve (Light off)				
Left Ear	2.19	0.88	0.67	0.37
Right Ear	2.58	0.82	0.32	0.43
Visual and Vestibular—ViVe (Light on)				
Left Ear	2.44	0.73	0.55	0.33
Right Ear	2.74	0.71	0.36	0.43
T-Sham (Light off)				
Left Ear	1.79	0.85	0.77	0.50
Right Ear	1.89	0.84	0.80	0.51
T-Sham (Light on)				
Left Ear	2.05	1.08	0.55	0.48
Right Ear	2.12	1.04	0.61	0.56
M-Sham (Light off)				
Left Ear	1.92	0.53	0.80	0.42
Right Ear	1.87	0.85	0.61	0.47
M-Sham (Light on)				
Left Ear	2.29	0.84	0.55	0.38
Right Ear	2.24	0.95	0.65	0.50

Mean and standard deviation for sensitivity and response bias in each experimental condition.

T-Sham = Thermal Sham stimulation; M-Sham = Mechanical Sham stimulation.

### 1. Differences between conditions in somatosensory sensitivity

No significant differences emerged between left ear NirCVS and right ear NirCVS in either Vestibular (t(9) = -1.428, p = 0.187) and Visuo-Vestibular conditions (t(9) = -1.109, p = 0.296) (Fig 1F). Data were therefore collapsed across ears in further analyses.

Somatosensory sensitivity was enhanced in all stimulation conditions relative to Baseline condition (Fig 1D). The enhancement in the combined Visuo-Vestibular condition was numerically greater than the changes in sensitivity observed in Visual and Vestibular conditions. To investigate these enhancements statistically, we compared each of these to zero. A Bonferroni correction was made for the three tests conducted, setting the significance level to 0.0167 two-tailed, but the p values are given here without correction. Data revealed a near-significant trend for enhancement of somatosensory sensitivity by visual stimulation (t(9) = 2.885, p = 0.018). Somatosensory sensitivity was significantly enhanced in both the Vestibular condition (t(9) = 3.946, p = 0.003) and in the multisensory Visuo-Vestibular condition (t(9) = 5.715, p<0.001) (Fig 1D).

Because our experimental method allowed only a small number of trials, there are upper and lower limits to d' estimates. Caution is needed to ensure that ceiling effects do not mask any putative superadditive modulation. We therefore tested whether a ceiling effect was present in our task, by contrasting the sensitivity estimates in the Visuo-Vestibular condition with a hypothetical perfect performance (a perfect score in our task would give d’ = 3.668). A significant difference was found (t(9) = -12.065, p<0.001). We thus believe that ceiling effects were not present in the task, leaving space for any putative superadditive interaction in the relevant experimental conditions.

To investigate the additivity of multisensory interactions, we directly tested a model in which the enhancement/suppression in Visuo-Vestibular condition is induced by a non-additive multisensory integration. We computed an index for multisensory integration, i.e. [Visuo-Vestibular—(Visual + Vestibular)], and we compared it to zero. We found a trend towards a significant under-additivity (t(9) = -2.144, p = 0.061), but this did not quite reach the conventional boundary of significance.

Because a model based on simple additivity of visual-somatosensory and vestibular-somatosensory effects could not be clearly rejected, we also used a Bayesian approach to evaluate the evidence for multisensory additivity. Specifically, we computed the Bayes factor (see http://www.lifesci.sussex.ac.uk/home/Zoltan_Dienes/inference/Bayes.htm for further details regarding the computation procedure), i.e. an index of the relative strength of our theory [45,46,47,48]. The Bayes factor is based on the principle that evidence supports the theory that most strongly predicted it, and it is estimated by comparing the (theoretical) alternative hypothesis to the null hypothesis. The Bayes factor can have a value ranging from 0 to ∞, where Bayes factor = 1 indicates the data are insensitive (i.e. do not favour one theory more than the other); Bayes factor >1 indicates the alternative is favoured over the null hypothesis and Bayes factor <1 indicate evidence for the null hypothesis over the alternative hypothesis. To compute the Bayes factor, we hypothesized that the unimodal sensory signals would combine following a half-normal distribution [45,46]. This assumption is warranted when effect sizes are likely to be small [45,46], as in both cross-modal visual-somatosensory [32,33] and vestibular-somatosensory [19,20,21] interactions. The likelihood of obtaining the data given the hypothesised multisensory integration model, i.e. [Visuo-Vestibular—(Visual + Vestibular)], is 0.13, while the likelihood of the null hypothesis is 0.24. The resulting Bayes factor of 0.54 is conventionally interpreted as plausible evidence for the null hypothesis of no interaction [45,46].

### 2. Differences between conditions in response bias

No significant differences emerged between left ear NirCVS and right ear NirCVS in either Vestibular (t(9) = 2.142, p = 0.061) and Visuo-Vestibular conditions t(9) = 1.309, p = 0.223). Data were therefore collapsed across ears in further analyses.

We found a tendency to respond “yes”, even when the shock was not present in all our experimental conditions, relative to Baseline condition (Fig 1). That is, although response bias was conservative overall, it was less so when a multisensory stimulus was present than when it was not. We tested whether these multisensory modulations of response bias were significant, by comparing response bias between experimental and baseline conditions. A significance level of 0.0167 two-tailed was adopted, reflecting a Bonferroni correction for three comparisons (Visual, Vestibular and Visuo-Vestibular conditions), but the p values are given here without correction. In line with previous reports, our data revealed a significant change in the response bias due to presence of a visual signal. Specifically, participants made more “yes” responses when the visual stimulus was present, than when it was not (t(9) = -4.264, p = 0.002) (Fig 1E). The bias in the Vestibular condition was also significantly different from the Baseline condition (t(9) = -4.142, p = 0.003), again because of an increased tendency to respond “yes” in the Vestibular condition. The multisensory Visuo-Vestibular condition (t(9) = -3.823, p = 0.004), showed the same result.

### 3. Comparisons between NirCVS and sham stimulation

To control for the specificity of the effects induced by NirCVS, we directly compared NirCVS to T-Sham and M-Sham stimulation (Fig 1G). Since no significant differences emerged between left ear and right ear T-Sham in both light absent condition (t(9) = -0.590, p = 0.569) and light present condition (t(9) = -0.196, p = 0.849), and no significant differences emerged between left ear and right ear M-Sham in both light absent condition (t(9) = 0.173, p = 0.866) and light present condition (t(9) = 0.112, p = 0.913), these data were collapsed for further analysis.

First, the changes in somatosensory sensitivity in light absent condition were estimated for NirCVS (i.e. Vestibular condition) and for both our sham stimulations. An unsurprising significant difference emerged between NirCVS and the T-Sham stimulation (t(9) = 3.820, p = 0.004). Similarly, NirCVS was significantly different compared to M-Sham stimulation (t(9) = 2.570, p = 0.030). No differences were found between T-Sham stimulation and M-Sham stimulation (t(9) = -0.353, p = 0.732). Second, the changes in somatosensory sensitivity in light present condition were estimated for NirCVS (i.e. Visuo-Vestibular condition) and for both our sham stimulations. A significant difference emerged between NirCVS and T-Sham stimulation even when the light was present (t(9) = 2.954, p = 0.016). Similarly, NirCVS was significantly different compared to M-Sham stimulation (t(9) = 3.080, p = 0.013). No differences were found between T-Sham stimulation and M-Sham stimulation when the light was present (t(9) = -0.783, p = 0.454). These results support a specific vestibular-induced modulation of somatosensory sensitivity.

We further estimated the changes in response bias in the light absent condition for NirCVS and both sham stimulations (T-Sham and M-Sham). Since no significant differences emerged between left ear and right ear T-Sham in light absent condition (t(9) = -0.315, p = 0.760) and light present condition (t(9) = -0.424, p = 0.682), and no significant differences emerged between left ear and right ear M-Sham in both light absent condition (t(9) = 1.726, p = 0.118) and light present condition (t(9) = -0.554, p = 0.593), these data were collapsed for further analysis.

A significant difference was found between NirCVS and T-Sham stimulation (t(9) = -3.451, p = 0.007), and M-Sham stimulation (t(9) = -2.919, p = 0.017) in light absent condition. Further, no differences were found between T-Sham and M-Sham stimulations in light absent condition (t(9) = 0.779, p = 0.456). A significant difference was found between NirCVS and M-Sham stimulation (t(9) = -2.323, p = 0.045) in light present condition. No significant differences emerged between NirCVS and T-Sham stimulation (t(9) = -1.413, p = 0.191) light present condition. Further, no differences were found between T-Sham and M-Sham stimulations when the light was present (t(9) = -0.144, p = 0.889).

## Discussion

No primary vestibular cortex has been identified in the primate brain [14]. Rather, vestibular inputs share the cortical projections of other sensory pathways, such as vision and touch [9,49]. Thus, anatomical links between vestibular input and other sensory modalities abound. Several physiological studies have considered the function of vestibular-visual links, but vestibular-somatosensory links remain relatively unstudied. Moreover, the existence, and possible functional significance of a three-way *vestibular-visual-somatosensory* interaction has rarely been considered. Multisensory neurons coding for vestibular, visual and somatosensory stimuli were found in the macaque VIP [13], homologous to the human vestibular areas in the posterior parietal cortex [14]. Bimodal neurons responding to vestibular and somatosensory stimulation were found in primate posterior parietal cortex (area 2v) immediately adjacent to primary somatosensory areas of hand and mouth [9,49]. Multisensory neurons coding for vestibular and somatosensory signals were also described in the PIVC and such neurons responded to vestibular stimulation of the semicircular canals and otolith organs, as well as touch applied on the arms, shoulders, neck, and legs. Similarly, neurons responding to visual and vestibular stimulation have been recorded in the fundus of the intraparietal sulcus, in the ventral and medial intraparietal areas (MIP) [13,50,51,52,53].

The existence of specialised neurons integrating vestibular and other inputs makes it unsurprising that vestibular, visual and somatosensory systems interact. In particular, vestibular-visual-somatosensory convergence has been described in almost all vestibular relays, including the vestibular nuclei, the thalamus and several areas in the cerebral cortex. However, the detailed form of this interaction has not been studied at a behavioural or systems level. Here we hypothesized that this interaction could involve trimodal multisensory enhancement, trimodal multisensory suppression, or independent bimodal multisensory influences of visual inputs on somatosensation [32,33,34,35] and of vestibular inputs on somatosensation [19,20,21]. We used detection of near-threshold somatosensory stimuli as a potential method for gauging the effect of multisensory visual and vestibular inputs on early somatosensory processes. Based on known anatomical projections between vestibular, visual and somatosensory regions, we hypothesised a number of possible models for multisensory interaction (Fig 1A, 1B and 1C), and we predicted their likely implications for somatosensory detection performance.

We found a near-significant trend for enhancement of somatosensory sensitivity by visual stimuli [32]. We also found a significant vestibular enhancement of somatosensory sensitivity. These findings replicate previous results [19], but importantly extend them to the novel NirCVS method of vestibular stimulation used. Our data in the trimodal vestibular, visual and somatosensory condition are clearly inconsistent with a multisensory enhancement model. Instead of enhancement, we found a trend towards under-additivity, with the combined vestibular and visual modulation of somatosensation being weaker than the sum of the two modulations independently. However, our data cannot conclusively rule out a simple additive model, since the trimodal effect did not quite reach the conventional boundary of statistical significance. In the additive model, the trimodal effect corresponds to the sum of the effects induced by bimodal visual-somatosensory and vestibular-somatosensory influences independently.

Previous anatomical studies demonstrated reciprocal inhibitory cortical interactions between visual and vestibular systems [24,25,26,27]. In particular, functional neuroimaging studies described activation of multiple cortical structures by means of vestibular stimulation, including the PIVC, the retroinsular cortex, the superior temporal gyrus (STG), the inferior parietal lobule (IPL), the precuneus, the anterior cingulum, and the hippocampus, accompanied by deactivations in the occipital and parietal visual areas [24,25,26,27]. This suppression may play a role in controlling eye movements and gaze stabilisation. Thus, if we assume that visual signals facilitate somatosensory sensation, and that vestibular stimulation inhibits visual signals, then an underadditive interaction between vestibular and visual processing should be present in our data. Specifically, vestibular stimulation should suppress the normal facilitation of touch by vision. We found a trend towards this pattern in our data.

The enhancement of somatosensory sensitivity by vestibular stimulation confirmed previous results [19,20,21]. Human neuroimaging studies showed that somatosensory cortical areas, as well as the insular cortex, were found to respond to vestibular and somatosensory inputs [11,12,18]. These studies provide a potential anatomical basis for the multisensory interaction found here. We thus suggest that vestibular inputs could act to enhance somatosensory sensitivity. One possible neural mechanism would be modulation of primary somatosensory cortical circuits by vestibular signals, along the lines previously proposed to explain visual enhancement of touch [54].

Caution is required in interpreting the non-significant difference that we found between left ear NirCVS and right ear NirCVS. Our data suggest that left ear and right ear stimulations have similar effects on somatosensory sensitivity. This lack of lateralization contrasts with previous findings using traditional cold caloric vestibular stimulation, which found stronger somatosensory effects following vestibular stimulation designed to activate the vestibular network in the right hemisphere (i.e. left cold CVS) [55,56]. Accordingly, neuroimaging studies identified the same asymmetry in the cortical vestibular system, suggesting that the cortical vestibular network is primarily located in the non-dominant right hemisphere in right-handed subjects [41]. However, these results have been collected in brain-damaged patients. The present data suggest that previous reports of vestibular hemispheric lateralisation might be related both to the unusual strong unilateral nature of traditional caloric vestibular stimulation and also to impaired cortical processing in the lesioned brain. In the present study, we found that NirCVS in healthy participants had similar effects on somatosensory sensitivity, irrespective of whether the left or right vestibular organs were stimulated.

Finally, our somatosensory detection task was confined to the left hand. We chose this condition because the strongest effects of vestibular stimulation have previously been found when stimulating the left vestibular organs, and thus the contralateral right hemisphere [56]. Methodological constraints due to NirCVS duration did not allow us to investigate possible changes in somatosensory sensitivity for the right hand. In particular, we applied the same precautionary principle with NirCVS as is generally applied to caloric vestibular stimulation studies: we performed no more than one stimulation per side per session. Given the limited window of NirCVS effectiveness, it was not possible to deliver somatosensory stimulation to both hands in the NirCVS conditions. Importantly, previous studies described bilateral enhancement of tactile sensitivity induced by vestibular stimulation [19]. Future, larger studies might factorially combine the side of vestibular and somatosensory stimulations, in multi-session experiments, to investigate this question more fully.

An additive effect of vestibular and visual signals on somatosensation could be explained by either of two alternative mechanisms. First, vestibular and visual signals could both influence somatosensation by a common but non-specific mechanism, such as arousal or attention. For example, the vestibular stimulation could increase arousal relative to baseline, and visual stimulation could also independently increase arousal. In particular, vestibular stimulation has been often associated with shifts of spatial attention. Vestibular-induced enhancement in sensitivity in some previous studies (though not the present one) depended on the side of vestibular stimulation, and thus on the hemisphere preferentially activated [20]. These hemisphere-specific effects cannot be explained by general arousal or changes in attention, since the peripheral vestibular organs receive comparable stimulation in both cases.

Alternatively, vestibular and visual stimuli might evoke separate and independent influences on the neural circuits responsible for somatosensory detection. For example, two independent projections, arising from vestibular and visual inputs, might converge in the somatosensory cortex, providing independent modulating influences. Behavioural and population-level measures cannot easily distinguish between multisensory convergence and multisensory modulation. However, there may be theoretical grounds for preferring a modulation account in this case. Recent theories of multisensory perception emphasise optimal integration of different channels of sensory information, by weighting each source according to reliability [57]. This integration aims at combining information about a spatio-temporally coherent *common source object* [57]. For example, two visual and somatosensory sensory channels may be combined to generate a perceptual representation of a single external object or event that is experienced both visually and tactually [58,59]. For instance, when manipulating objects, the object's size can be judged simultaneously by vision and touch. Information from these two modalities should therefore be integrated in the nervous system to benefit from their redundancy [58]. Integrating the information from the two modalities yields a more certain estimate of an object’s size, compared with the noisy estimate from each modality alone [58].

In contrast, the “common source object” concept that underlies these studies may not readily apply to the trimodal situation studied here. The vestibular system may not describe an external physical object or source in the same way that visual or haptic exteroception do. Instead, modern accounts characterise the vestibular system as representing self-motion: when moving the head, visual, vestibular, and somatosensory signals all give rise to an estimate of the heads' position and orientation. Hence, the sensory system integrates these signals into a coherent representation of bodily position. However, such experience is rather different compared with the perceptual representation of an external object [58,59]. Further, our vestibular stimulation was spatially and temporally distinct from our somatosensory stimuli, and occurrences of all three stimuli (vestibular, somatosensory and visual) were statistically independent. Therefore, the concept of converging multisensory information used in previous multisensory studies may not readily apply in this case. Instead, our modulations of somatosensory detection could be considered as probing the state of the corresponding early “unimodal” cortex, prior to the stage at which spatiotemporally coherent source objects are bound from multiple sensory inputs. We suggest that the vestibular influence on somatosensory sensitivity may be an example of modulation of early somatosensory cortex via the anatomical projections identified by previous vestibular stimulation studies.

In conclusion, we investigated interactions between vestibular, visual and somatosensory inputs in a simple somatosensory detection task. We rejected a model of multisensory interaction based on supra-additive multisensory enhancement. Rather, we found additive influences of vestibular and of visual signals on somatosensory processing, or even a trend towards underadditivity. This trend is consistent with a model in which visual and vestibular inputs have additive, facilitatory influences on somatosensory processing, while vestibular inputs additionally suppress visual processing. These perceptual results are consistent with known neuroanatomical interactions between these three sensory systems.

## Supporting Information

S1 Fig EOG recordings. Examples of raw EOG data.
(TIF)
