# Supplementary figures and images for: Multisensory Interactions between Vestibular, Visual and Somatosensory Signals
Source: PLoS One. 2015 Apr 13;10(4):e0124573. doi: 10.1371/journal.pone.0124573 (PMC4395320; doi:10.1371/journal.pone.0124573)

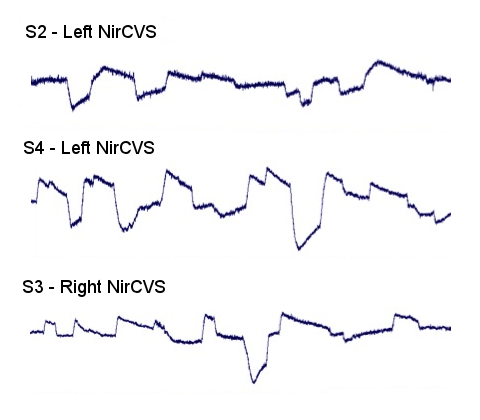

Supplement: S1 Fig — Examples of raw EOG data. (TIF) [file pone.0124573.s001.tif]
